# Excitation Intervals Enhance Performance in Perovskite Solar Cells

**DOI:** 10.1021/acsami.5c18736

**Published:** 2025-10-18

**Authors:** Sarah C. Gillespie, Jarla Thiesbrummel, Veronique S. Gevaerts, L. J. Geerligs, Jeroen J. de Boer, Gianluca Coletti, Erik C. Garnett

**Affiliations:** † LMPV-Sustainable Energy Materials Department, 55952 AMOLF Institute, Science Park 104, Amsterdam 1098XG, The Netherlands; ‡ 2859 TNO Department Solar Energy, Westerduinweg 3, Petten 1755LE, The Netherlands; § School of Photovoltaic and Renewable Energy Engineering, 7800 University of New South Wales, Sydney, New South Wales 2052, Australia; ∥ University of Amsterdam, Science Park 904, Amsterdam 1098XH, The Netherlands

**Keywords:** halide perovskites, perovskite solar cells, photoluminescence, ion migration, stability, dark recovery

## Abstract

Halide perovskites face intrinsic stability challenges primarily due to light- and bias-induced ion migration. To mitigate ion-mediated degradation on operationally relevant time scales, this work investigates how introducing brief periodic intervals of light and darkness (LD cycling) can stabilize the average efficiency of perovskite films and devices. Systematic photoluminescence (PL) studies reveal that dark intervals on the order of seconds significantly suppress nonradiative recombination and slow degradation. The extent of PL enhancement depends on the duration of the dark time, the material composition, and critically, the sample’s age. Remarkably, LD cycling increases PL by more than 7-fold even in aged samples that would otherwise undergo photodarkening under continuous illumination. Moreover, the PL kinetics under LD cycling mirror the corresponding open-circuit voltage dynamics in full solar cells, showing that local emission changes provide a direct measure of device-level behavior. Device measurements similarly show that LD cycling enhances the power conversion efficiency compared to continuous illumination and mitigates deterioration over extended operation. This strategy highlights a potential pathway to dynamically preserve or even improve perovskite performance in future optoelectronic applications.

## Graphical abstract

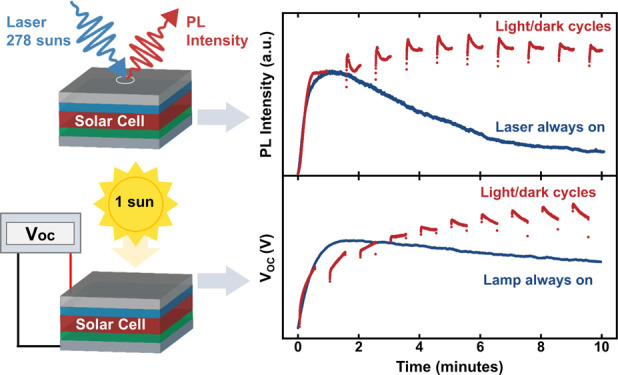

## Introduction

Metal halide perovskite semiconductors have huge potential in photovoltaics (PV), with a certified record device efficiency currently standing at 27.3%.^1^ Their impressive performance stems from excellent optoelectronic properties, including long charge-carrier lifetimes and diffusion lengths.^1–5^ However, they typically also contain high densities of mobile ionic defects which, under even moderate illumination conditions or electrical bias, can migrate through the film, consequently altering the electronic properties over time.^6–10^ These optoelectronic changes are particularly evident in the complex dynamics observed in photoluminescence (PL) time-series measurements.^4,11–16^ The variability in perovskite PV performance has also prompted extensive discussion on how to accurately measure the efficiency of devices, including preconditioning steps and measurement scan rates.^17,18^

Recently, it has been shown that under standard test conditions for perovskite PV cells, ion accumulation at the perovskite surface leads to a reduction in the carrier extraction efficiency and can induce degradation in the device.^19^ Despite this, computational studies indicate that the open-circuit voltage (*V*_OC_) is higher with mobile ions present than if the ions were absent, due to their positive effect on the energy level alignment within the device.^20–22^ Thus, the power conversion efficiency ultimately depends on the interplay between these ion-induced opposing effects.

Even with some ion-induced performance deterioration during a day of illumination, devices can (albeit not always) recover their initial performance after being stored in the dark overnight.^23–28^ This day/night recovery concept has resulted in renewed discussions on how to fairly assess the true degradation rate in perovskite solar cells compared to the standard stability tests based on crystalline silicon PV technology.^29,30^ Here, however, we do not address the various methods used to restore devices to their full performance between day and night. Rather, we focus on harnessing the light-responsive behavior of ions to yield an enhanced average power output over the course of a single day. Specifically, we report that dynamic illumination conditions—controlled intervals of light and darkness—can drive the perovskite into an optimized state. In this state, PV performance can be maximized without compromising the device stability. The enhanced quasi-equilibrium state is sustained only due to the alternating cycles, and once the cycling is removed, the device performance deteriorates as anticipated. The results are corroborated with systematic PL studies, which further elucidate how dynamic light/dark (LD) cycling enhances the performance as a function of perovskite composition, dark time, sample age, and more. These findings indicate that introducing unconventional light-harvesting protocols during the day, for example by incorporating switchable glass onto the device or by periodically tilting the module, could potentially mitigate the ion-induced degradative effects at the interface, thereby addressing some of the key remaining stability issues pertaining to perovskite solar cells.^31–33^

## Results

### Photoluminescence Results

We begin by presenting the PL time series observed for our triple-cation, mixed-halide perovskite films with a chemical composition of Cs_0.07_(FA_0.8_MA_0.2_)_0.93_Pb­(I_0.8_Br_0.2_)_3_, hereafter termed the 80:20 composition. All films were fabricated on glass substrates and encapsulated in 60 nm of electron-beam-evaporated SiO_2_; fabrication procedures are described in the Experimental Section. Figure 1a shows a representative normalized PL time series acquired from the 80:20 sample during an excitation sequence alternating between illumination and darkness; Figure S1 presents the corresponding resolved PL spectra together with the time-series evolution of the PL peak wavelength over the same temporal range. Excitation was provided by a focused 405 nm continuous-wave laser set at an intensity of 220 W/cm^2^ (2784 suns equivalent). At the start of the illumination, the PL initially exhibited an exponential decay, with a decay constant of τ_decay,1_ = 0.72 ± 0.01 s. Approximately 10 s into the light soaking, the PL started to recover at a rate of 0.34%/s (relative to the starting value); then, from 50 s onward, the PL continued to improve more slowly at a rate of 0.07%/s. This dynamic behavior is typical for perovskite thin films and is attributed to several competing processes, most likely involving the initial formation of light-activated electronic traps, followed by a slow trap-annihilation process involving recombination of iodide vacancies and interstitials.^4,10,11,28,34,35^ When excitation was removed for 5 min and subsequently reapplied to the same spot, the PL intensity at the start of the second illumination sequence (indicated by the blue marker) was 2.02 times higher than at the end of the previous light soaking period (red marker). Even after the exponential decay (τ_decay,2_ = 7.64 ± 0.02 s), the PL intensity remained at least 1.14 times higher than the PL maximum during the first illumination period. This “dark-induced” enhancement was found to be reproducible across multiple cycles, as demonstrated by the continually enhanced third and subsequent LD periods.

**Figure 1:**
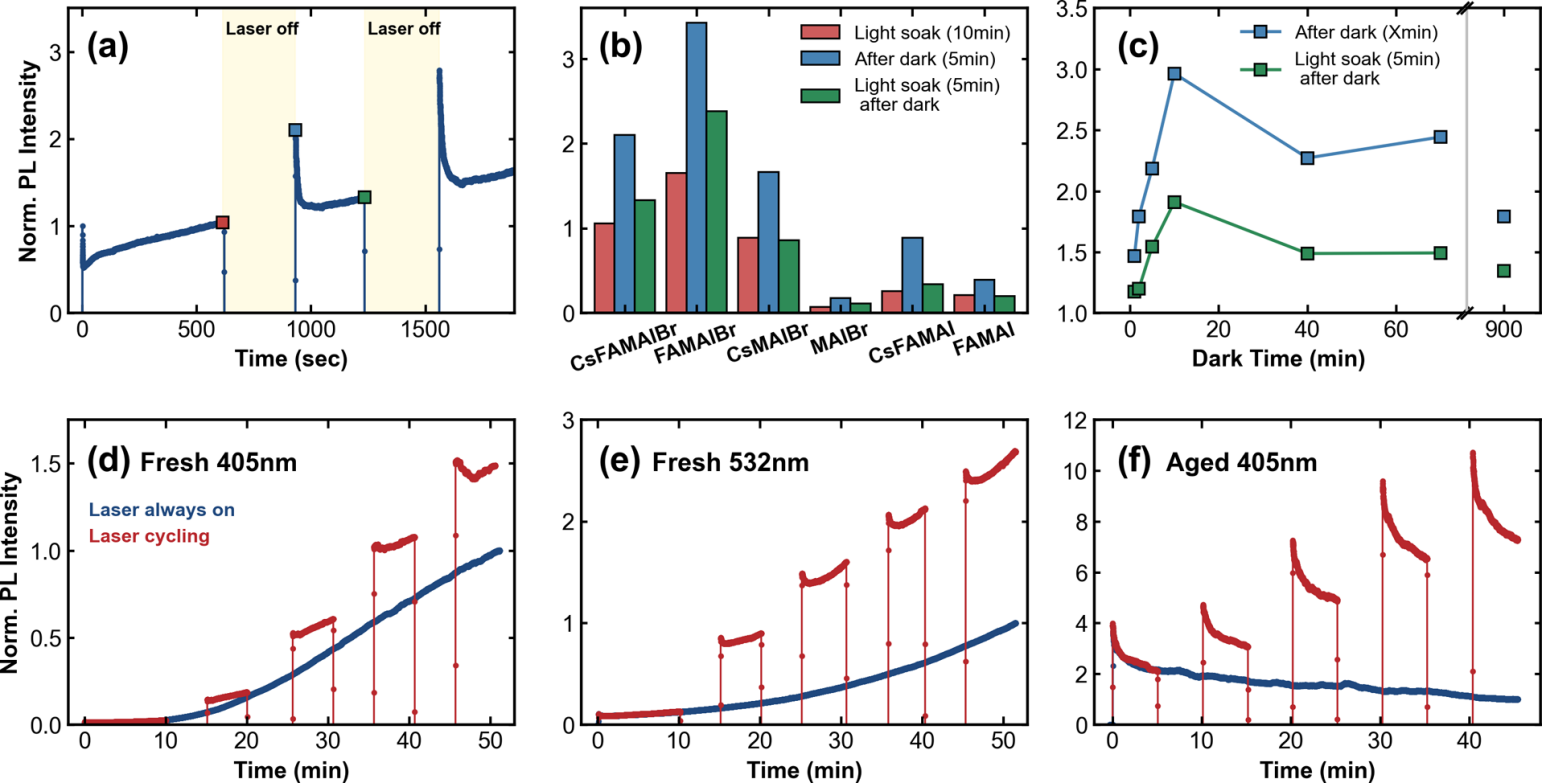
(a) Representative PL trace obtained from a SiO_2_-encapsulated triple-cation, mixed-halide perovskite film (80:20 composition). (b) Comparative PL signals of the six perovskite compositions investigated in this work with the bar chart values normalized to the PL at the start of the first illumination sequence. The *x*-axis labels show the chemical abbreviations for each composition, which are listed in full in Table S1. (c) PL enhancement of the encapsulated 80:20 film as a function of dark time, where the blue points signify the PL immediately after re-excitation, similar to the blue marker shown in (a). The green points show the PL after five min of continuous illumination following the dark period. (d) Comparative PL time series measured under continuous excitation and under LD cycling. The sample was fabricated 1 day prior to measurement. (e) The same comparative PL time series but using 532 nm excitation. (f) PL time series measured on the same sample shown in panel (d) but acquired after 3 weeks of dark storage in N_2_. Panels (a)–(c) were measured at a 2784 suns-equivalent illumination, while panels (d)–(f) were measured at a reduced 1114 suns-equivalent illumination. Measuring under different intensities enabled us to elucidate the relative importance of applied intensity compared with other factors, such as the dark time duration.

The LD enhancement effect was not limited to 80:20 perovskite composition. Among the five additional films tested in this work, all showed improved PL after a 5 min dark period between illumination, which is illustrated by comparing the blue and green bars to the red bars in Figure 1b. The complete time series is shown in Figure S2a. This effect was observed regardless of whether the sample photobrightened or photodarkened during the initial 10 min of illumination as indicated by the relative positions of the red bars in the chart. These observations imply that the mechanism driving the LD enhancement is not simply a continuation of any light-activated photobrightening or photodarkening processes and therefore cannot be explained by any light-only mechanisms described in the literature.^4,11,28,36^

The magnitude of this LD PL enhancement depends not only on composition but also on the illumination intensity (Figure S2b), the interface applied (Figure S2c,d), and importantly, the duration of the dark times. As shown in Figure 1c, the PL enhancement was maximized after a 10 min dark interval, while the reversibility of the LD enhancement—the rate at which the sample returned to its initial state—was found to be significantly slower than its onset. Even after keeping the sample in the dark for 15 h, the PL remained higher at the start of its second illumination period than at the end of its first illumination period. In Figure 1c, the PL signal was normalized to its value at the end of the initial 5 min light soaking to simplify the comparison. The blue curve represents the integrated PL measured immediately after the sample was illuminated, while the green curve represents the PL signal 5 min into the second light soaking period after the indicated dark time. To ensure that perovskite memory effects did not interfere with the experiment, the measurements for different dark times were all performed on a fresh spot on the sample.^37,38^ Notably, the long-term elevated PL was to some extent found to occur on samples that were exposed to full-field illumination and left in the dark for 12 h (Figure S3a). This test was conducted with a blue, unfocused LED (10 mW/cm^2^) and performed entirely under nitrogen conditions, further indicating that the dark enhancement is not the result of any atmospheric reactions.^12,13,39–41^

### LD Cycles Enhance Perovskite PL

Based on the initial PL findings, we next considered whether the rate of LD enhancement mirrored that of light-only enhancement. We compared the continuous and LD PL time series for a freshly prepared 80:20 sample under reduced illumination conditions (88 W/cm^2^, 1114 suns equivalent). This comparison was conducted 1 day after fabrication, with the sample stored in the dark under nitrogen between fabrication and measurement. Figure 1d depicts the continuous illumination (blue) and LD cycling (red) signals in which the PL was normalized to the end value of the continuous series. Despite having received a reduced total photon dose, the PL intensity in the LD cycling measurement was 50% higher after 50 min than in the continuous illumination case. A similar enhancement trend was observed using a focused 532 nm laser at comparable sun-equivalent intensity (Figure 1e). The relevance of the excitation energy is that the LD enhancement does not seem to be related to any selective PbI_2_ formation process, as previously shown to occur at high excitation energies in MAPbI_3_ films.^36^

Importantly, after 3 weeks of dark nitrogen storage, the sample photodarkened when exposed to continuous illumination (blue curve, Figure 1f). Comparing the continuous curves in Figure 1d,f is significant; the only difference between these measurements is the age of the sample. This highlights that the dynamic PL response is potentially most critically influenced by the sample history, in addition to known variables such as excitation energy, excitation intensity, and applying pulsed rather than continuous excitation.^11,12,36,42^ Even under inert conditions with a fully encapsulated sample, intrinsic material changes significantly affect the PL response over time. This behavior may be related to factors such as strain relaxation, evaporation of volatile molecules at the edges of the film, or changes in the Urbach tail states.^43–45^ Nevertheless, despite the change from photobrightening to photodarkening, applying LD cycles still resulted in a 7.3-fold PL enhancement at the final point of the measurement (red curve, Figure 1f), further emphasizing that this dark-driven enhancement is independent of the light-driven changes.

### Correlating PL with Device Performance

Building on our initial findings that local PL in perovskite films can be enhanced by LD cycling, we next investigated the effects of such LD cycles on complete perovskite solar cells with a slightly different composition; see the Experimental Section for device fabrication details and measurement procedures. We compared the local PL changes of devices with the changes in their *V*_OC_. During the device’s PL measurement, as illustrated in Figure 2a, the PL was collected from a 113 μm^2^ focused area on the sample, under 405 nm laser illumination at an intensity equivalent to 278 suns. The order-of-magnitude reduction in the applied illumination in the device case compared with the films was to verify that the applied intensity was not the dominant reason for general PL trends. In contrast, the *V*_OC_ time series was measured at 1 sun using a simulated AM1.5G spectrum (Figure 2b). Both PL and *V*_OC_ were monitored under periodic cycling: 30 s of illumination followed by 30 s of darkness, except for a 1 min continuous illumination period at the start of the PL experiment. These cycles are colored red in Figure 2c,d, while the blue curves represent the continuous illumination measurements. Over a short 10 min period, both the PL and *V*_OC_ exhibited closely correlated dynamics. Both signals rose within the first minute, followed by a decay under continuous illumination. The accelerated degradation may be partially attributed to the interfacial reactions at the C_60_ interface, as shown in Figure S3b. Under LD cycling, both the PL and *V*_OC_ continued to increase, surpassing the continuous illumination maxima. In the second half of the experiment, both signals consistently exhibited some exponential decay, albeit with different decay constants. Upon averaging the final three cycles in both cases, τ_decay,*PL*_ = 5.3 ± 0.2 s and τ_decay,*V*_OC__ = 27.4 ± 0.6 s. Interestingly, the PL decay constant of the device is similar to that of the pristine film measured earlier, despite the roughly 10-fold lower excitation intensity applied to the full cell.

**Figure 2:**
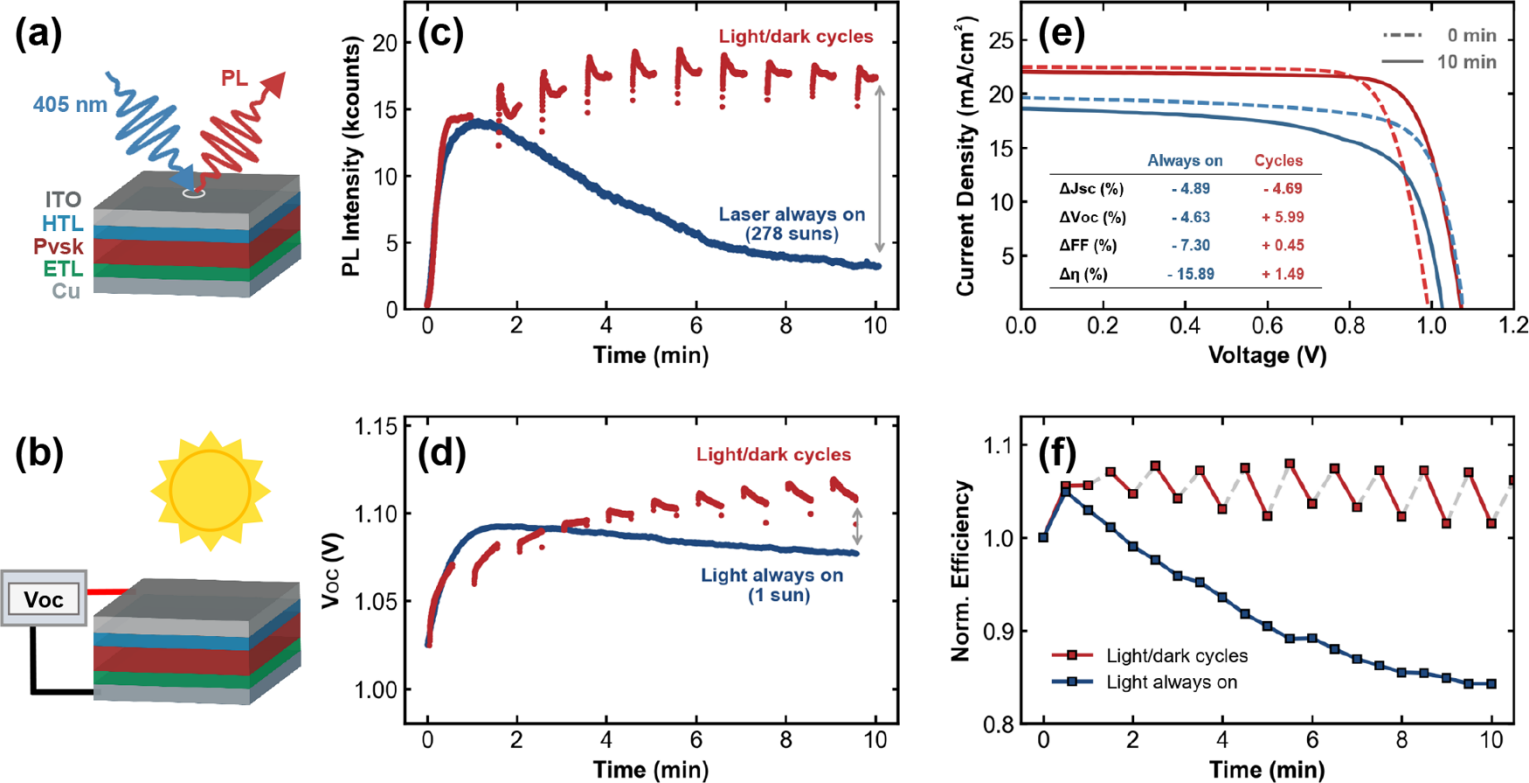
(a), (b) Schematics illustrating the measurement procedures for obtaining the solar cell’s PL and *V*_OC_. Panels (c) and (d) show the corresponding PL and *V*_OC_ time series under continuous illumination (blue) and LD cycling (red). (e) Representative *JV* curves comparing 10 min of continuous illumination (blue) with periodic 30 s LD cycles (red). The absolute differences in *JV* parameters arise from the pixel-to-pixel variation. The dashed lines in both plots represent the starting curve, while the solid line represents the *JV* curve after the 10 min experiment. Inset: table presenting the percentage change of the *JV* parameters for the always-on and cycled experiments. (f) Corresponding cell efficiency time series normalized to *t* = 0 min, highlighting the efficiency trends for the always-on (blue) and cycling (red) experiments.

To quantitatively compare the differences between the solar cell’s PL and measured *V*_OC_, we use the established relation that linear differences in *V*_OC_ values correspond to logarithmic changes in PL:^46^

$$\Delta V_{\mathrm{OC},\mathrm{PL}}=V_{\mathrm{therm}}\ln\left(\frac{{\mathrm{PL}}_{\mathrm{cycl}}}{{\mathrm{PL}}_{\mathrm{cont}}}\right)$$ 1

where PL_cycl_ and PL_cont_ are the PL intensities under LD cycling and continuous illumination, respectively. Using this equation, the PL-derived difference at the end of the experiment in Figure 2c (gray double arrow) is Δ*V*_OC,PL_ = 44.8 mV. By comparison, the directly measured Δ*V*_OC_ = 39.6 mV at the gray double arrow in Figure 2d. The fact that these trends coincide despite the PL being performed under a substantially higher excitation density is striking. The nearly identical kinetics of the degradation highlight that localized high-intensity PL captures the same underlying processes as full-device *V*_OC_ measurements and can serve as a reliable proxy for tracking device performance.

The magnitude of *V*_OC_ enhancement was found to depend on the duration of the dark time (Figure S4), mirroring the dependence shown in Figure 1c. The correlated PL-*V*_OC_ trends signify that the LD-driven enhancement is not the result of mobile defects diffusing out of the laser spot, thereby resulting in a local PL improvement.^4^ Moreover, as shown in Figure S4, the *V*_OC_ continues to improve even after tens to hundreds of seconds of dark time—time scales comparable to the diffusion of mobile ionic species in perovskite films.^35,47–51^ We therefore suggest that the correlated LD enhancement is most likely driven by an interfacial defect-mediated mechanism involving the diffusion of mobile ions across the sample and their recombination at the surface. This is corroborated by the fact that, without appropriate passivation, the PL tends to be interface-limited in perovskite devices.^4,16,52,53^ Even with the higher surface recombination from the C_60_ contact in our measured half-stack, the PL still enhanced under LD cycling (Figure S2).^54,55^ This overall hypothesis is further validated by the full-field LED measurements discussed earlier in this work.

Extending to additional device parameters, we subsequently compared *JV* curves between devices that were illuminated continuously at 1 sun and held at *V*_OC_ between *JV* sweeps (ISOS-L1) and devices that underwent 30 s LD cycling for 10 min. Holding at *V*_OC_ between sweeps was based on community recommendations and was intended to further accelerate any rate of performance deterioration in the device.^24,30^ A representative comparison between these curves is shown in Figure 2e, while the tracked change in device efficiency is presented in Figure 2f. Comparisons among all four device parameters are shown in Figure S5. By reading the table inset in Figure 2e, the short-circuit current density, *J*_SC_, in both instances was reduced by approximately the same amount, while the *V*_OC_ in the LD cycling case was improved compared to the continuous illumination case, which agrees with the *V*_OC_ tracking comparison. This ultimately led to a moderate overall efficiency enhancement in the cycling case. With a separate device, we repeated the 30 s LD cycling for 10 min, then measured the *JV* curves under continuous illumination for an additional 10 min to determine the steady-state changes following the brief cycling period. As shown in Figure S6, the performance remained stable during the 10 min of cycling but deteriorated over the subsequent 10 min without dark intervals, emphasizing that dark intervals are necessary to maintain the enhanced quasi-stable state.

We then compared devices under extended LD cycling conditions, with light and dark times resembling the time scales previously considered in Figure 1. The normalized device parameters of this extended test are compared in Figure 3. In this case, the *V*_OC_ enhancement trend was found to be similar to that previously observed, while the *J*_SC_ on average tends to decrease in the LD cycle at an average rate similar to the continuous illumination reference. Notably, the *J*_SC_ values measured immediately after the dark times were enhanced compared with their values at the end of the previous light soak. This reveals a correlated enhancement between all four parameters and importantly signifies that the *V*_OC_ enhancement does not come with a trade-off in extraction efficiency, as may be the case if the LD enhancement mechanism was due to the formation of an inert, passivating layer at the interface. Symmetric devices with Au/Cr lateral contacts also showed reduced resistance and enhanced current under cycling conditions (Figure S7), where the current enhancement was found to depend on the distance between the electrical contacts. Ultimately, this means that both carrier recombination can be temporarily reduced, and carrier extraction can be temporarily enhanced if a dark interval on the time scale of seconds is introduced between illumination periods.

**Figure 3:**
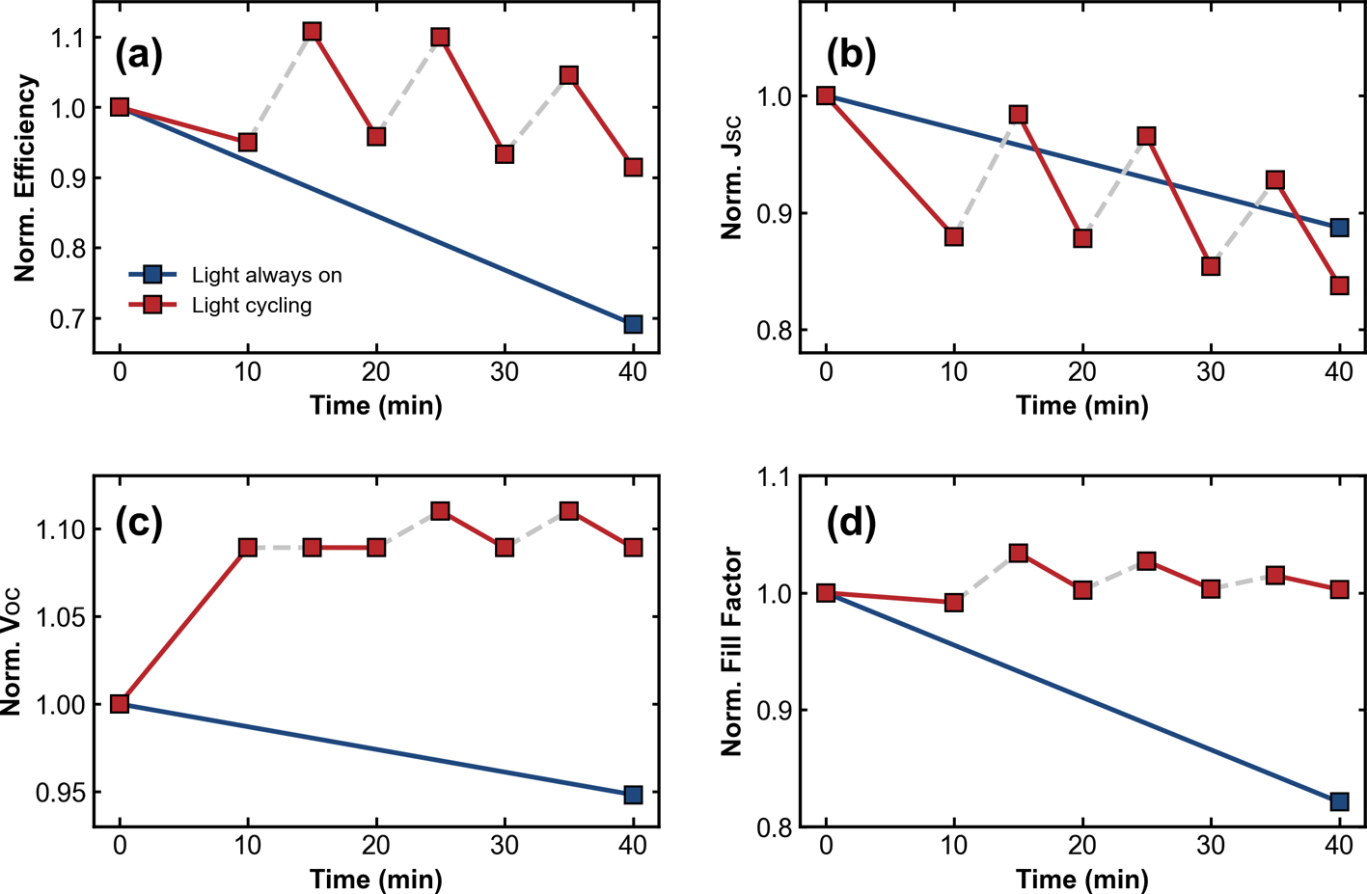
Normalized (a) efficiency, (b) *J*_SC_, (c) *V*_OC_, and (d) fill factor (FF) are shown for a 40 min comparison between continuous illumination (blue) and LD cycling (red). The gray dashed lines represent the times at which the light was removed during the LD cycling measurement.

### Stabilizing Photovoltaic Performance

With the combined short-term device enhancement introduced, we next consider whether this LD cycling could mitigate the short-term device degradation known to occur over several hours of continuous illumination.^23,25–29,56–58^ In Figure 4a, we show the measured efficiency of our reference cell under continuous light soaking conditions for more than 5 h. The data points indicate the times at which a full *JV* sweep was measured, and the blue curve represents the combined exponential and linear fit that accurately model the efficiency trend within this time frame. Similar to the earlier tests, all devices were held at *V*_OC_ between *JV* sweeps. All four device parameters over time are shown in Figure S8. The efficiency decreases exponentially in the first 10 min of illumination and then deteriorates linearly for the remaining time. This trend was reproducible across different samples (Figure S9), which enabled us to confidently apply the same fitting approach to extrapolate the likely linear degradation rates for devices that underwent LD cycles. In Figure 4b, we show the efficiency results for a device that alternated between 39 min of continuous illumination and 39 min of LD cycling (with the continuous illumination regions shaded in light gray). All four device parameters from this measurement are presented in Figure S10. During the asymmetric LD cycles, the device was kept in the dark for 30 s after every 270 s of illumination. Between continuous illumination and LD cycling, the device was kept in the dark for 60 s to separate these periods and to “reset” the device to a state that was close to its starting performance. In Figure 4b, the red line represents the measured efficiency output, while the dashed black line represents the expected continuous illumination degradation rate if the device had been measured under continuous illumination. The periods of constant illumination between LD cycling exhibited steady efficiency deterioration, confirming that the trends observed within the LD cycles were due to the introduced brief dark times and were not attributable to some other device effect. Even with the rapid decays that occurred during the light intervals within the dynamic LD cycles, all three cycles at 40, 120, and 200 min exhibited reduced overall degradation rates compared to the extrapolated linear curve. In fact, in the second and third cycles, the device efficiency even increased during the 39 min, emphasizing that by introducing even brief dark times, LD recovery can outcompete light-induced degradation and therefore stabilize the overall time-averaged efficiency output.

**Figure 4:**
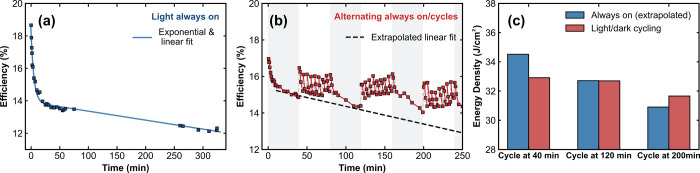
(a) Representative efficiency data for the devices studied in this work. These devices were measured under continuous 1 sun illumination conditions at AM1.5G at room temperature and indoors. The blue markers represent the points at which the *JV* sweeps were collected with the combined exponential and linear fit shown by the blue line. (b) Second representative device efficiency curve plotted for alternating continuous illumination and asymmetric LD cycling periods. The red line and markers follow the actual measured data, with the black dashed line indicating the anticipated efficiency trend if the device had been continuously illuminated for the entire experiment. The gray background highlights when continuous illumination was applied, compared to the LD cycle periods (white background). (c) Total collected energy density for the three LD cycles from panel (b) (red). The corresponding hypothetical energy collected under continuous illumination, determined from the extracted fit over the same time periods, is shown in blue.

We recognize that while there may be some relevance in stabilizing overall device performance using LD cycles, introducing dark time is only impactful if the average power output during these dynamic cycles is greater than the average power output from continuous illumination. This calculation should account for both the difference in degradation rates between the illumination conditions and the ≈10% dark time, where no power is extracted at all during the LD cycles. In Figure 4c, we illustrate this comparison by plotting the integrated energy density harvested during the three LD cycles presented in Figure 4b. In blue, we additionally show the hypothetical total energy density that would be collected if the device did not undergo LD cycling. Comparing the results for the cycle at 40 min, the continuous illumination case is clearly preferable over LD cycling. However, the LD cycles in the second and third rounds resulted in either the same or greater electricity produced than that in the continuous illumination case, respectively. These findings suggest that introducing brief periodic intervals during which the device receives no sunlight over the course of a full day may result in both enhanced and stabilized overall device performance. We repeated the comparative LD experiment with longer dark intervals (60 s of dark time every 240 s) and with a higher frequency of dark intervals (30 s of dark time every 120 s); in both cases, we found that the dark-induced enhancement could not outcompete the extended time during which no energy could be collected. This indicates that the optimal percentage of dark time necessary within the LD cycles is somewhere below 20%.

We emphasize that while these measurements were performed on aged devices (to mimic real-world conditions in which PV modules are likely to be installed several weeks or months after fabrication), these are still preliminary findings and were tested only under indoor measurement conditions. Further research is necessary to elucidate the LD impact under real-world operating conditions, such as extended outdoor testing on different device structures, coupled LD cycling with day/night cycles, under various temperatures, humidity, and intensity levels.^25,30,59,60^

## Discussion

We now consider possible mechanisms underlying the enhanced efficiency and PL observed with LD cycling, although we emphasize that mechanistic follow-up studies are needed for full validation. We first reiterate mechanisms that we have already ruled out. Because the samples were encapsulated and some measurements were further performed under nitrogen, atmospheric effects are unlikely to dominate the LD enhancement.^12,13,39–41^ The comparison between excitation wavelengths (405 versus 532 nm) further indicates that passivating species such as PbI_2_ are not responsible for the dark enhancement.^36^ Finally, the enhancement cannot be attributed to mobile defect migration out of the illuminated region, as similar effects were observed under both localized and full-field illumination.^4^

Instead, we propose that all PL and device changes are surface-driven. Our previous lifetime analyses of these materials support this: the bulk of the examined triple-cation, mixed-halide perovskite is of high quality (τ_bulk_ = 2 μs), whereas recombination losses are concentrated at the surfaces, which pin the PL.^54^ Moreover, the time scales of the strongest enhancements—both in light and dark—align with mobile ion dynamics. In our recent work, we measured a characteristic ionic diffusion time of 77 s in a 620 nm film, which directly altered the PL on that time scale, implying interfacial ionic reactions as a central driver for PL changes.^35^

Interestingly, the degradation and recovery kinetics resemble those reported by Nie and co-workers,^28^ who attributed photocurrent degradation to light-activated polaronic trap states at the perovskite interface. They showed that 1 min in the dark was sufficient to deactivate these metastable traps, restoring *J*_SC_ without strongly affecting *V*_OC_. However, trap deactivation alone cannot explain enhancements beyond the initial performance, nor the correlated improvements in both *V*_OC_ and PL reported here.

We therefore propose that LD enhancement arises from a combination of ion migration and interactions between mobile ions and metastable trap states. Under illumination, ions may accumulate at the interface alongside polaronic traps, reducing carrier extraction and increasing nonradiative recombination.^19,28,61^ Once the light is removed, the traps deactivate rapidly (within 1 min), while slower-moving ions redistribute. It is possible that under illumination, polaronic defects screen ionic Frenkel pairs (vacancy–interstitial pairs), preventing their recombination.^4,10^ In the dark, these pairs may rearrange and recombine, lowering the interfacial defect density. The redistribution time scales match the dark durations needed for maximum enhancement (Figures 1c and S4).^35,49,50,62^

The combined reduction in defect density and deactivation of trap states would then yield the correlated increases in PL, *V*_OC_, and *J*_SC_ upon reillumination. Repeated illumination could further drive ionic defects to the surface, while dark periods allow recombination, progressively reducing nonradiative losses over successive cycles (Figure 2c,d). This is effectively an optical zone refining process where LD cycles anneal out interfacial defects. While this mechanism is consistent with our film and device observations and supported in part by the trap deactivation dynamics identified by Nie and co-workers, we emphasize that the specific role of ion migration remains speculative, and alternative interpretations should not be excluded.

In addition to mechanistic validation, further investigation is needed to determine whether these enhancements can be permanently “locked-in”—that is, whether it is possible to prevent degradation once the enhanced state from LD cycling has been reached. This concept of inducing a permanent change may be achievable through recrystallization processes or through the incorporation of specific additives whose properties can be activated by laser or thermal annealing after fabrication.^63–68^ Such strategies could potentially stabilize the beneficial ionic configurations established during LD cycling with the goal of permanently forming a favorable energy landscape that suppresses detrimental ion migration and preserves device performance.

On a practical note, while engineering a complete system to implement LD cycling in perovskite solar cells is beyond the scope of this work, the integration of such a dynamic approach may not be as complex as one might expect. For example, if LD enhancement remains effective even under partial shading, then periodic module tilting could be employed to help maintain the enhanced state.^69,70^ Alternatively, with the development of smart glass—glass capable of selectively switching between transparent and opaque states—and its current applications in optimizing light and heat regulation within buildings, extending its application to perovskite PV is a justifiable consideration.^31,71^ Standard smart glass typically requires approximately 1 W/m^2^ to maintain the opaque state and requires nearly zero power input for the transparent state; therefore, the additional energy consumption would remain below 1% if integrated into a high-efficiency module.^72^ Notably, several studies have explored utilizing the dynamic properties of halide perovskites themselves for direct application as smart glass materials.^73–75^ However, methods for stabilizing the average energy output from such dynamically operated modules, as well as the full integration costs, remain critical considerations for this hypothetical scenario. Finally, if the LD cycling mechanism could be replicated using a fully electrical approach rather than an optical one, then it may be possible to retain the benefits of the induced enhanced state without reverting to additional dynamic layers or mechanical tilting.

## Conclusions

In conclusion, we demonstrated that localized high-intensity PL measurements can quantitatively reproduce device-level degradation trends. Systematic PL analyses revealed that LD cycling substantially enhances the emission compared to continuous excitation, with the magnitude of enhancement depending on dark time, composition, and interface. Moreover, an LD enhancement was observed under both localized and full-field excitation. Importantly, PL changes correlated directly with changes in the open-circuit voltage (Δ*V*_OC_ ≈ 40 mV). From this analysis, we established a simple LD protocol for complete perovskite devices under 1 sun conditions that yielded a higher energy output compared with continuous excitation.

Finally, we emphasize that after more than ten years of research into various stabilization strategies, perovskites remain entirely unconventional dynamic semiconductors. Their material properties continually evolve over time due to ion-induced reactions and other reactions, which are ultimately intrinsic to the material itself. It is therefore worth considering whether dynamic solutions are the key to maintaining a stabilized photovoltaic performance. Beyond PV, if the ionic response in perovskites could be dynamically controlled then these materials could even be exploited for novel applications, such as artificial synapses, adaptive materials, and more.^76–79^ The simple consideration of dynamic LD cycling explored in this work yielded enhanced short-term results, though we argue that dynamic biasing, heating, or a combination of these parameters could ultimately result in enhanced material performance without compromising stability for applications in perovskite photovoltaics and beyond.

## Experimental Section

### Fabrication Details

All materials were purchased from suppliers outlined in the materials sections of our previous works.^35,54^ The perovskite films were prepared on glass substrates. The glass was scrubbed with 1% Hellmanex III solution in deionized (DI) water, then sonicated for 15 min in 70 °C water, then for 15 min in acetone, and finally for 15 min in isopropanol. The glass was dried using N_2_ and treated under UV-ozone for 30 min.

To prepare the perovskite films, 1.24 M solutions of PbI_2_ in 4:1 DMF:DMSO and PbBr_2_ in 4:1 DMF:DMSO were stirred overnight at 70 °C. A 1.5 M solution of CsI in DMSO was stirred at 70 °C for 2 h. The PbI_2_ solution was added separately to FAI powder and to MAI powder to form 1.24 M FAPbI_3_ and 1.24 M MAPbI_3_, respectively. Similarly, the PbBr_2_ solution was added separately to FABr powder and to MABr powder to form 1.24 M FAPbBr_3_ and 1.24 M of MAPbBr_3_. These perovskite solutions were then each stirred again at 70 °C for a further 2 h before they were combined in the appropriate ratios, along with the CsI solution, to form the six compositions tested in this work. These compositions are given in Table S1.

Before spin coating, all solutions were cooled to room temperature and filtered with a 0.45 μm PTFE filter. The solutions were spin coated at 4000 rpm for 30 s after a 6 s ramp-up time and were quenched 15 s before the end of the spin coating using 170 μL of chlorobenzene. All samples were annealed at 100 °C for 30 min before being encapsulated in 60 nm of SiO_2_ using a Polyteknik Flextura M508E electron-beam evaporator, where the evaporation rate was set to 0.06 nm/s.

For the interface comparison shown in Figure S2c,d and S3b, 25 nm of C_60_ and 7 nm of 2,9-dimethyl-4,7-diphenyl-1,10-phenanthroline (BCP) were sequentially evaporated over the complete 80:20 film before either encapsulated further with 60 nm SiO_2_, as described above, or left unencapsulated (for the comparison shown in Figure S3b).

Lateral devices were fabricated on glass substrates with a UV lithography lift-off procedure using a MA-N1410 resist. The resist was UV-exposed on a Süss MA6/BA6 mask aligner and then developed in MA-D533/s. A 5 nm Cr adhesion layer and 80 nm Au electrode material were deposited on the patterned resist by e-beam physical vapor deposition using the same evaporator listed above, both with a rate of 0.05 nm/s. Lift-off was performed by soaking in acetone for 1 h. The substrates were treated under a 1 min oxygen plasma treatment before the perovskite solution of composition Cs_0.07_(FA_0.83_MA_0.17_)_0.93_Pb­(I_0.83_Br_0.17_)_3_ was spin coated on top of the substrate and contacts. The fabrication and spin coating of this perovskite follow similar procedures to what was described above. All lateral devices were encapsulated in 60 nm of SiO_2_.

For the complete solar cells, the patterned ITO substrates were cleaned by sonication for 10 min, first in acetone, then in 3% Hellmanex III solution with DI water, then in DI water only, and finally in isopropanol. They were treated using oxygen plasma for 4 min. Poly­[bis­(4-phenyl)­(2,4,6-trimethylphenyl)­amine] (PTAA) was applied as the hole transport layer; the PTAA solution (1.75 mg/L in toluene) was spin coated onto the ITO at 6000 rpm for 30 s, following a ramp-up time of 3 s, and then annealed at 100 °C for 10 min. After cooling to room temperature, PFN-Br­(poly­(9,9-bis­(3′-(N,N-dimethyl)-N-ethylammonium-propyl-2,7-fluorene)-*alt*-2,7-(9,9-dioctylfluorene))-dibromide) (0.05 mg/mL in methanol) was deposited on top of the PTAA layer dynamically at 4000 rpm for 30 s. A 1.2 M Cs_0.07_(FA_0.83_MA_0.17_)_0.93_Pb­(I_0.83_Br_0.17_)_3_ perovskite solution, with a 10% excess PbI_2,_ was prepared by similarly preparing the FAPbI_3_, MAPbBr_3,_ and CsI solutions (although the perovskite solutions were stirred at room temperature overnight). The perovskite was spin coated on top of the hole transport layer at 4000 rpm for 400 s, following a ramp time of 3 s. Ten s into the spinning, the perovskite was quenched with 300 μL of ethyl acetate. The samples were then annealed at 100 °C for 1 h. Twenty-five nm of C_60_, 8 nm of BCP, and 100 nm of Cu were thermally evaporated on top of the perovskite through a mask to complete the devices.

### Measurement Details

Photoluminescence measurements for all cases except for the full-field LED illumination test (Figure S3a) were performed by using a WITEC alpha300 SR confocal imaging microscope coupled to a Thorlabs S1FC405 405 nm CW diode laser. The PL was collected in reflection mode with a focused laser spot size of 113 μm^2^. For the 532 nm excitation experiments, the WITEC system was instead coupled to a 532 nm Nd:YAG laser. Spectra were acquired using a charge-coupled device (CCD) and spectrometer, and the PL intensity was determined by integrating across the PL peak.

For the full-field PL experiment, PL was collected in transmission mode. A 465 nm LED (Cree LED) was used as the excitation source and was set to an illumination intensity of approximately 10 mW/cm^2^. An encapsulated 83:17 perovskite film was placed directly above the LED, and a Thorlabs 650 nm FEL long-pass filter was used to block the LED illumination from being detected by the silicon detector. The PL was monitored as the photocurrent generated by a BPW34 silicon photodiode (OSRAM, Infineon Technologies). The full-field PL experiment was conducted entirely within a N_2_-filled glovebox.

All electrical measurements were performed using an Agilent B2902A Source-Measurement Unit, and the AM1.5G illumination was applied with an Oriel SOL2 94062A (6 × 6) Class ABA (Newport) solar simulator. For the *JV* curves, the voltage sweep rate was set to a high speed of 1 V/s to minimize hysteresis effects and reduce any influence from the measurement itself.

## Supplementary Material



## Notes

Perovskite composition table; supplementary analysis; and supporting figures (PDF)
