# Supplementary material for: Excitation Intervals Enhance Performance in Perovskite Solar Cells
Source: ACS Appl Mater Interfaces. 2025 Oct 18;17(43):59476–85. doi: 10.1021/acsami.5c18736 (PMC12581118; doi:10.1021/acsami.5c18736)
Supplement: Supplementary file 1 [file am5c18736_si_001.pdf]

Supporting Information
Excitation Intervals Enhance Performance in Perovskite Solar Cells

Sarah C. Gillespie,[†,‡] Jarla Thiesbrummel,[†] Veronique S. Gevaerts,[‡] L.J. Geerligs,[‡] Jeroen J. de Boer,[†] Gianluca Coletti,[¶] and Erik C. Garnett[*,†,§]

†*LMPV-Sustainable Energy Materials Department, AMOLF Institute, Science Park 104, Amsterdam, 1098XG, The Netherlands*

‡*TNO Department Solar Energy, Westerduinweg 3, Petten, 1755LE, The Netherlands*

¶*School of Photovoltaic and Renewable Energy Engineering, University of New South Wales, Sydney, New South Wales 2052, Australia*

§*University of Amsterdam, Science Park 904, Amsterdam, 1098XH, The Netherlands*

E-mail: e.garnett@amolf.nl

Table S1: List of the six perovskite compositions studied by PL in this work, together with their chemical abbreviations, as referenced in Figure 1b in the main text.

**Perovskite Composition**	**Abbreviation**
$Cs_{0.07}(FA_{0.8}MA_{0.2})_{0.93}Pb(I_{0.8}Br_{0.2})_3$	CsFAMAIBr
$FA_{0.8}MA_{0.2}Pb(I_{0.8}Br_{0.2})_3$	FAMAIBr
$Cs_{0.07}MA_{0.93}Pb(I_{0.8}Br_{0.2})_3$	CsMAIBr
$MAPb(I_{0.8}Br_{0.2})_3$	MAIBr
$Cs_{0.07}(FA_{0.8}MA_{0.2})_{0.93}PbI_3$	CsFAMAI
$FA_{0.8}MA_{0.2}PbI_3$	FAMAI

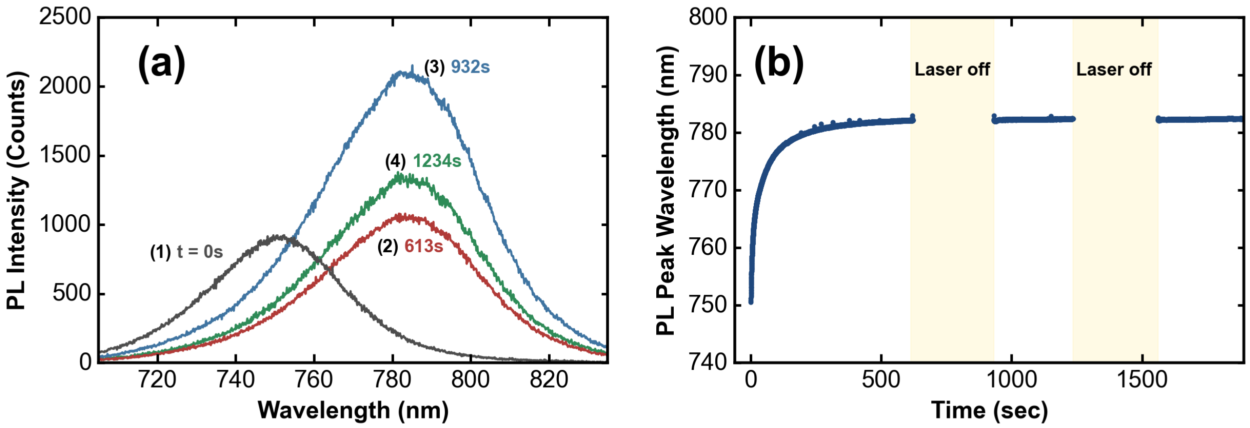

Figure S1: (a) PL spectra corresponding to the PL time series shown in Figure 1a. Each spectrum is color-coded to match the markers in Figure 1a. (b) Evolution of the PL peak energy over time, obtained from Lorentzian fits to the spectra.

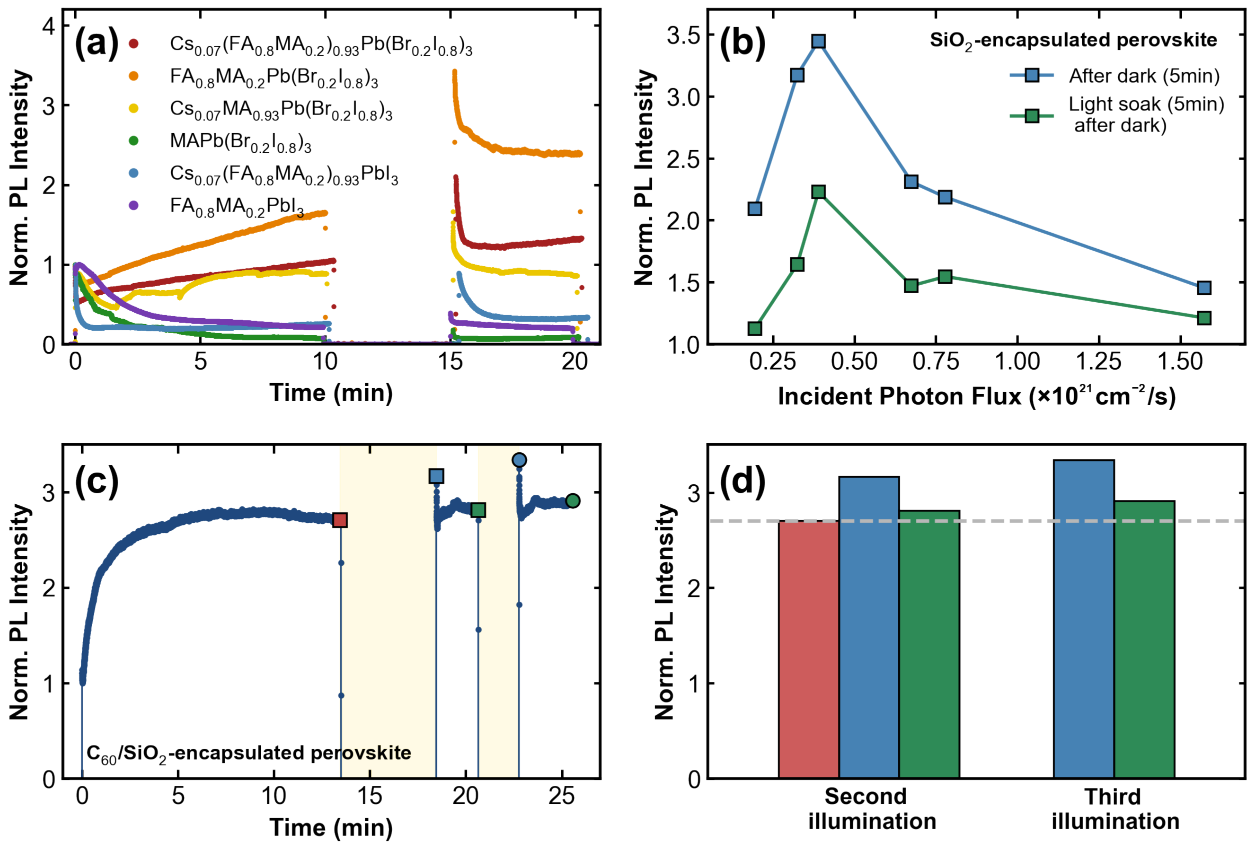

Figure S2: (a) PL time series of the six different perovskite compositions measured under 405 nm excitation at the same illumination intensity of 88 W/cm^2. (b) The measured PL of the 80:20 sample under different illumination intensities. In this panel, the PL is normalized to the end of the preceding light-soaking sequence (10 minutes of light soaking). The sample was kept in the dark for 5 minutes between illumination periods. The blue markers represent the PL immediately after re-excitation, while the green markers represent the PL following the second 5-minute light soak. (c) PL time series for an 80:20 sample with a 25 nm film of C_{60} and a 7 nm layer of BCP between the perovskite and SiO_2. (d) Bar chart highlighting the PL values of the square markers (second illumination period) and round markers (third illumination period) shown in panel (c).

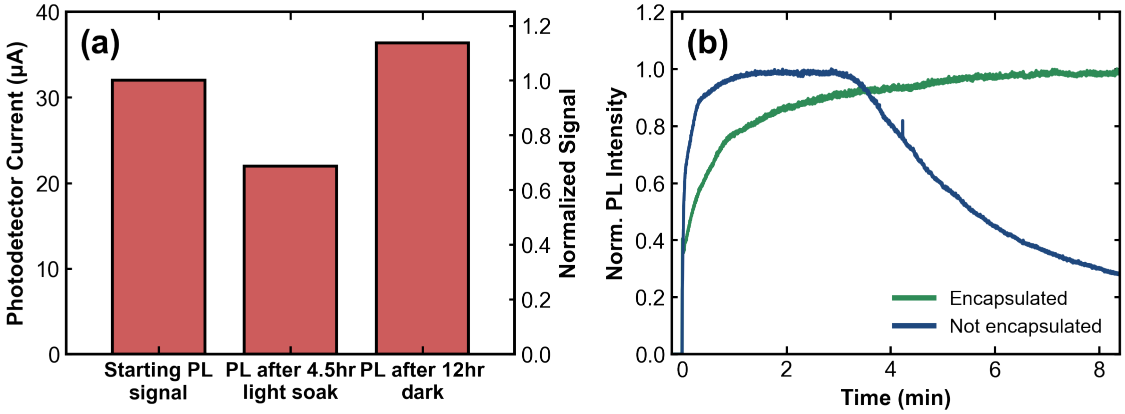

Figure S3: (a) Bar chart showing full-field PL as measured using a silicon photodetector. The absolute current signal is shown on the left side of the panel, and the normalized values (relative to the starting PL signal) are shown on the right side of the panel. After an extended illumination time of 4.5 hours, the full-field PL signal decreased, as shown by the middle bar. However, the full-field PL signal was enhanced to a level above the starting PL after allowing 12 hours in the dark. (b) Comparison between measuring the C_{60}-contacted film (previously shown in Figure S2c, in green) with a sample with C_{60} and BCP but not encapsulated in SiO_2 (blue).

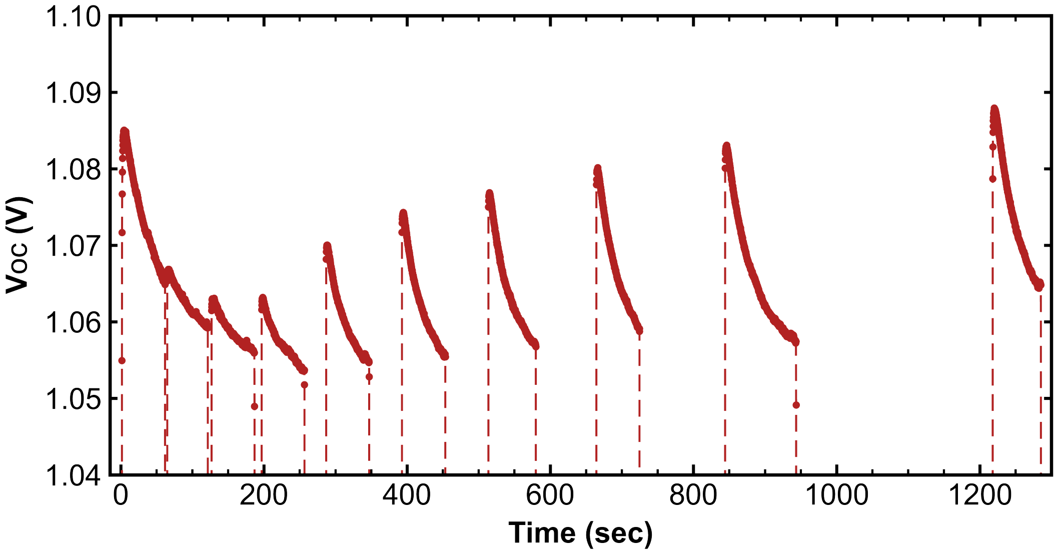

Figure S4: Time series of the open-circuit voltage, V_{OC}, for sequentially increasing dark times applied between 1-minute intervals of 1 sun equivalent AM1.5G illumination. Unlike the previous PL measurements, this time series was performed continuously on the same sample. The approximate duration of each dark interval was as follows (in chronological order): 3 seconds for the first two cycles, then 10 seconds, 30 seconds, 40 seconds, 60 seconds, 85 seconds, 120 seconds, and finally 260 seconds. Notably, the V_{OC} exhibits a steady improvement with increasing dark time.

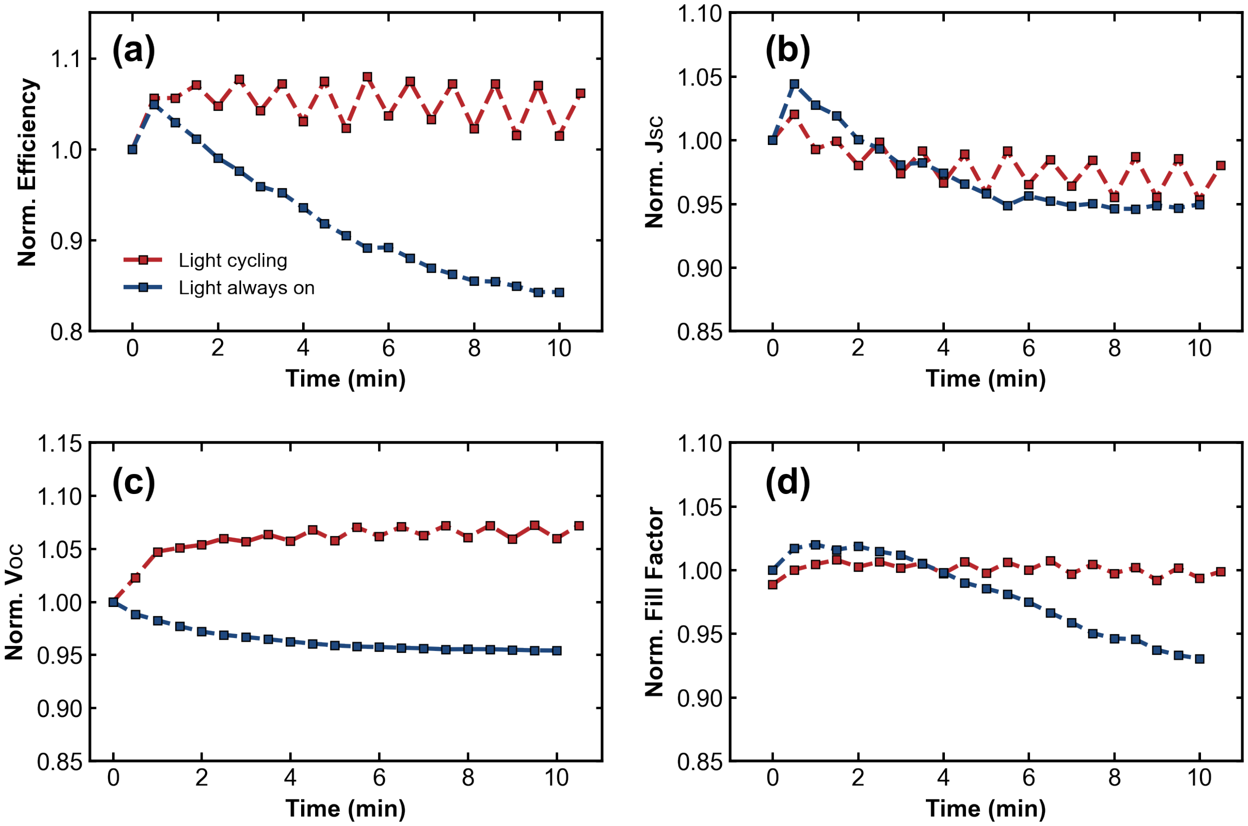

Figure S5: The complete device parameter comparison between the devices shown in Figures 2e and 2f, where (a) is the normalized power conversion efficiency, (b) the normalized J_{SC}, (c) the normalized V_{OC}, and (d) the normalized fill factor. The blue data represent the case where light was continuously applied, while the red data show the case in which light was periodically cycled between 30 seconds of illumination and 30 seconds of dark.

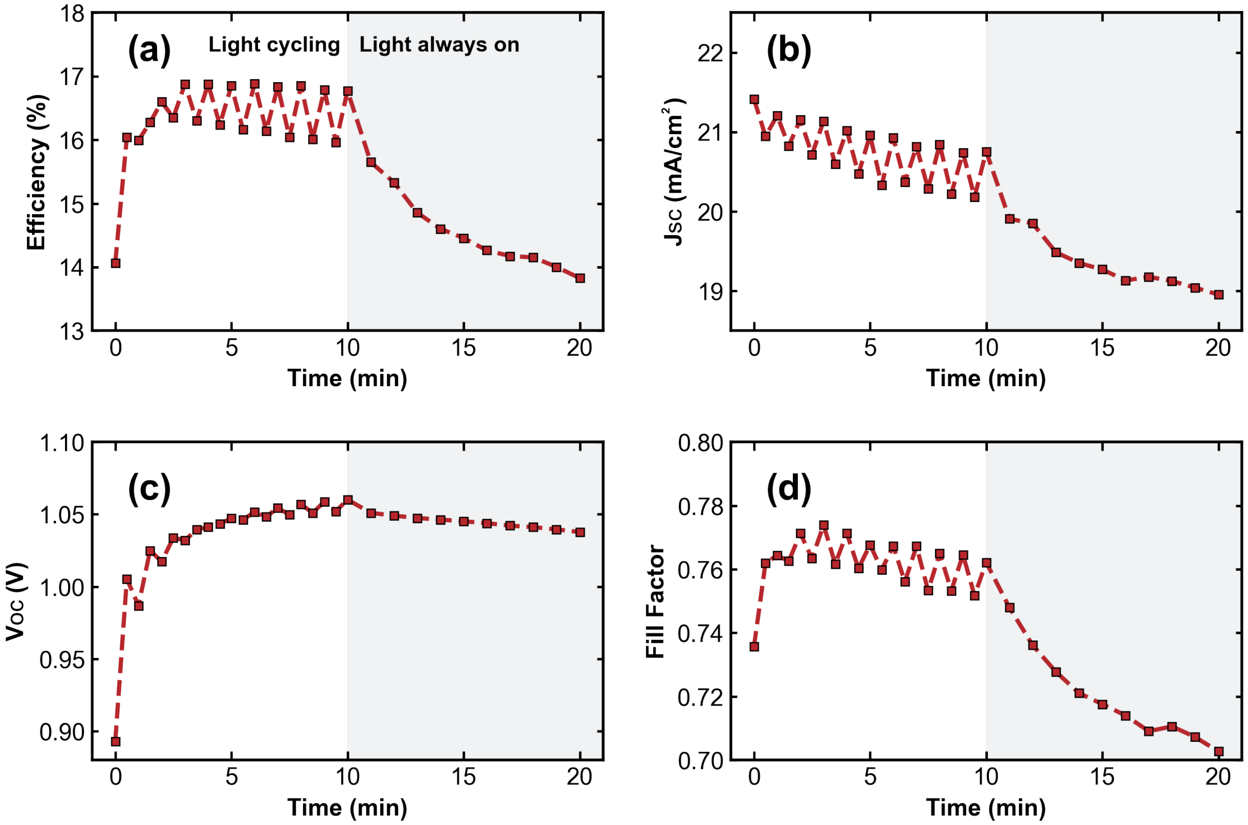

Figure S6: (a) The measured power conversion efficiency, (b) J_{SC}, (c) V_{OC} and (d) fill factor, over a 20 minute experiment. The device was exposed to periodic LD cycles (30 seconds of light then 30 seconds of dark) for the first 10 minutes (white background), then continuously exposed under constant 1 sun illumination for the subsequent 10 minutes (gray background).

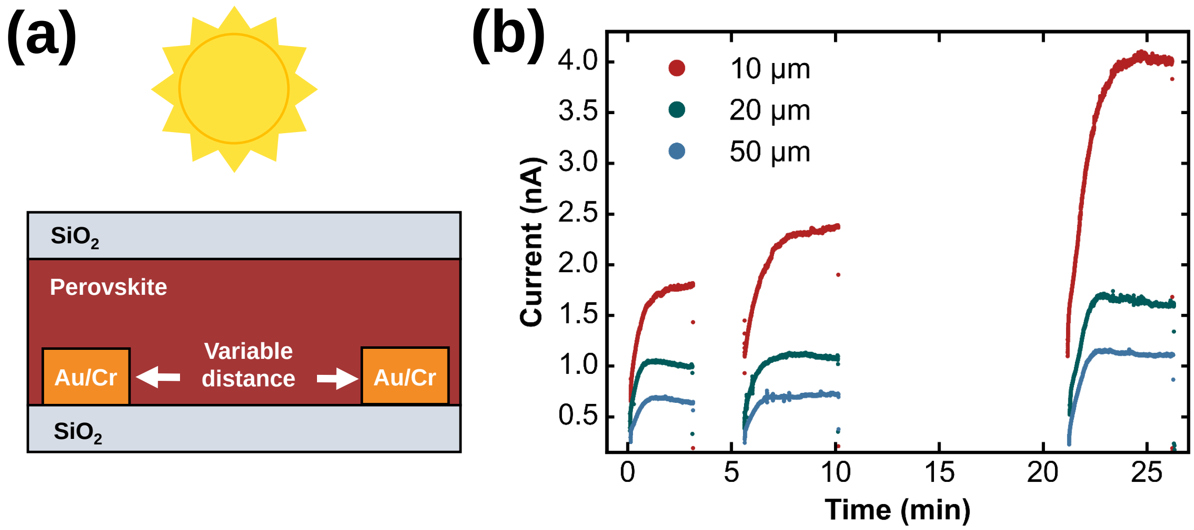

Figure S7: (a) Schematic of the symmetric lateral contact device measured. The distance between the lateral electrodes was defined by the evaporation mask, with values of either 10 µm, 20 µm, or 50 µm. The electrodes were 100 nm thick, and the 83:17 perovskite layer was approximately 600 nm thick. The excitation was applied at 1 sun equivalent with an AM1.5G solar spectrum. A 20 mV voltage bias was applied between the electrodes, and the current was measured. (b) The measured current for different samples with different lateral distances between the electrodes under an LD cycle. In all cases, the current was enhanced after the dark interval. Notably, the relative enhancement was highest for the sample with the smallest distance between the electrodes (10 µm, shown in red).

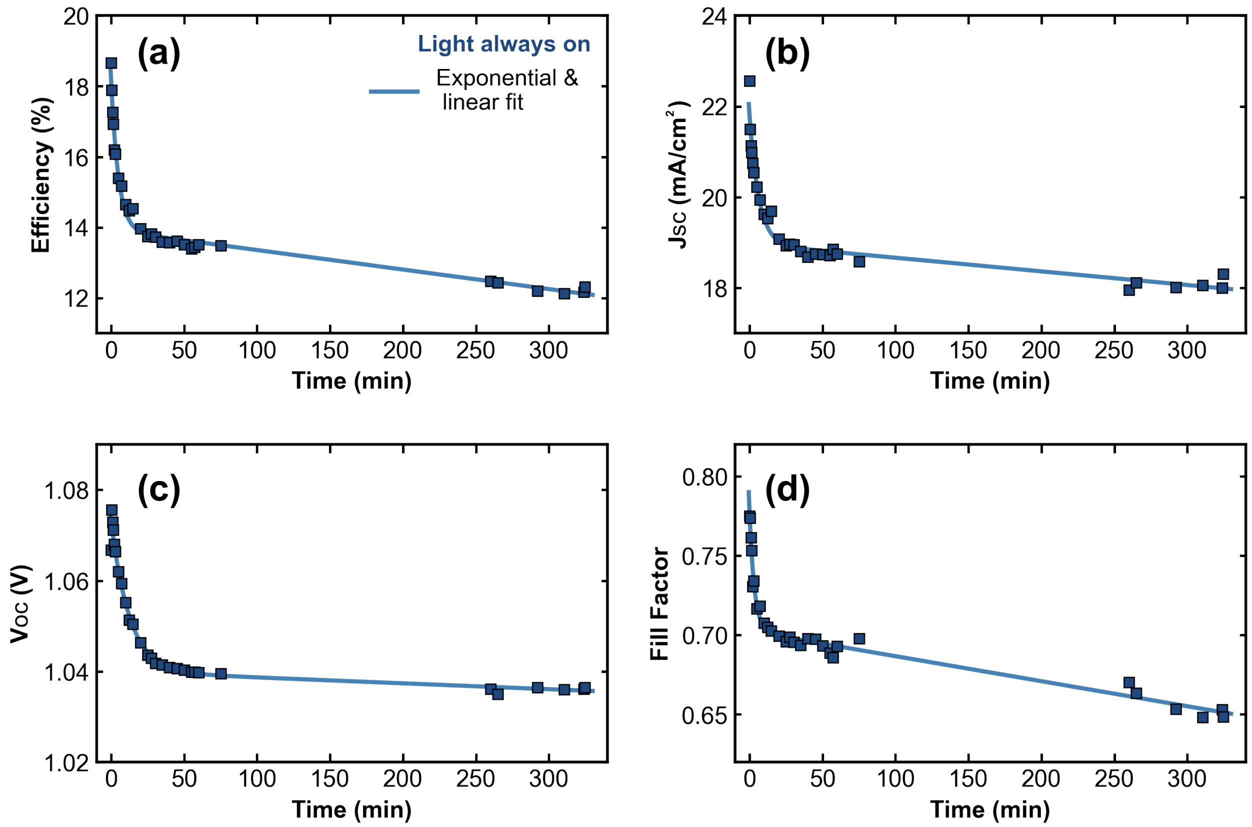

Figure S8: (a) Power conversion efficiency, (b) J_{SC}, (c) V_{OC}, and (d) fill factor time series under continuous illumination. All four of these parameters correspond to the device efficiency shown in blue in Figure 4a. A combined exponential and linear fit was used to accurately model all four parameters, although the rates of degradation varied between them.

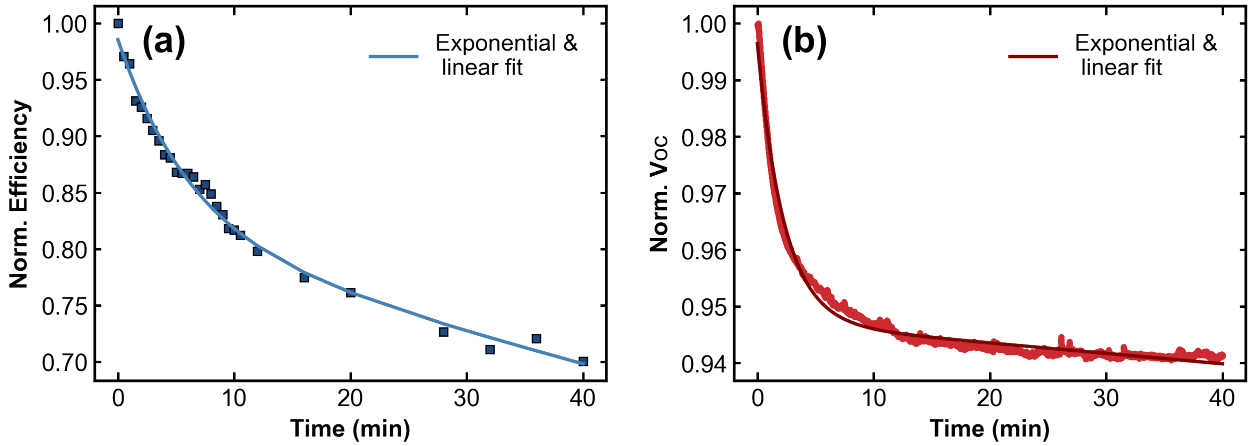

Figure S9: Supporting measurements indicating that a combined exponential and linear fit can empirically model the JV degradation trends. $Y = A\exp[-t/\tau] + Bt + C$, where Y is the JV parameter, t is time, and τ, A, B, C are the fit parameters. Panels (a) and (b) show the normalized efficiency and V_{OC} data for different devices. The solid curves represent the empirical fits.

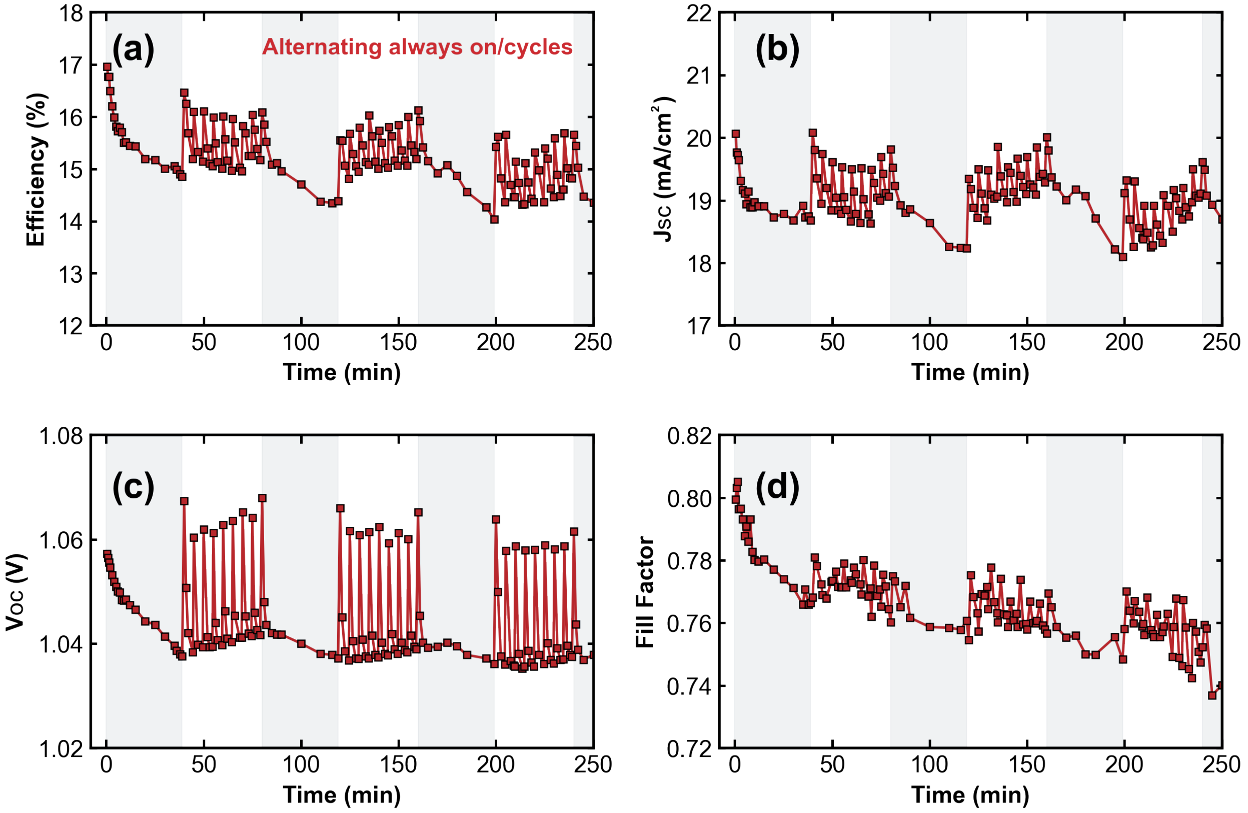

Figure S10: All four device parameter time series for the device that alternated between continuous illumination and LD cycling as shown in red in Figure 4b. (a) The power conversion efficiency, (b) J_{SC}, (c) V_{OC}, and (d) fill factor. Notably, a 30-second dark time enabled a significant enhancement in V_{OC}, consistent with the PL enhancement measured in this work. The J_{SC} also increased immediately after the dark periods, while the fill factor did not significantly vary. The continuous illumination periods are highlighted with the gray background, compared to the white background for the LD cycle periods.
